# Upregulation of Neuronal Rheb(S16H) for Hippocampal Protection in the Adult Brain

**DOI:** 10.3390/ijms21062023

**Published:** 2020-03-16

**Authors:** Gyeong Joon Moon, Minsang Shin, Sang Ryong Kim

**Affiliations:** 1 BK21 plus KNU Creative BioResearch Group, School of Life Sciences, Kyungpook National University, Daegu 41566, Korea; neobios7@gmail.com; 2 Brain Science and Engineering Institute, Kyungpook National University, Daegu 41566, Korea; shinms@knu.ac.kr; 3 Department of Microbiology, School of Medicine, Kyungpook National University, Daegu 41944, Korea

**Keywords:** Rheb(S16H), neurotrophic interaction, neurotrophic factor, Alzheimer’s disease

## Abstract

Ras homolog protein enriched in brain (Rheb) is a key activator of mammalian target of rapamycin complex 1 (mTORC1). The activation of mTORC1 by Rheb is associated with various processes such as protein synthesis, neuronal growth, differentiation, axonal regeneration, energy homeostasis, autophagy, and amino acid uptake. In addition, Rheb–mTORC1 signaling plays a crucial role in preventing the neurodegeneration of hippocampal neurons in the adult brain. Increasing evidence suggests that the constitutive activation of Rheb has beneficial effects against neurodegenerative diseases such as Alzheimer’s disease (AD) and Parkinson’s disease (PD). Our recent studies revealed that adeno-associated virus serotype 1 (AAV1) transduction with Rheb(S16H), a constitutively active form of Rheb, exhibits neuroprotective properties through the induction of various neurotrophic factors, promoting neurotrophic interactions between neurons and astrocytes in the hippocampus of the adult brain. This review provides compelling evidence for the therapeutic potential of AAV1–Rheb(S16H) transduction in the hippocampus of the adult brain by exploring its neuroprotective effects and mechanisms.

## 1. Introduction

Neurodegenerative diseases, which are becoming increasingly prevalent, are characterized by the physical decay and eventual loss of neurons. Neurodegenerative changes within the hippocampus and an extended neuronal network involving the medial temporal and medial parietal lobes result in the memory impairment observed in Alzheimer’s disease (AD) [1,2]. AD is the result of slow neuron degeneration, which starts in the hippocampus and spreads to the rest of the brain. The hippocampus plays a strategic role in the neurobiology of memory, being involved in the hierarchical spread of amyloid and neurofibrillary tangles [3,4]. The World Health Organization has identified dementia caused by AD as a public health priority, which will affect an estimated 115 million people worldwide by 2050 if there is no effective preventative strategy. Despite progress in elucidating the underlying mechanisms and genes involved in certain hallmarks of AD, including neuronal degeneration, extracellular neuritic plaques, and intracellular neurofibrillary tangles, the mechanisms underlying the degeneration and functional disruption that occur in AD remain unknown, and treatments to prevent the progression of the disease are limited [1,5,6]. Although the incomplete understanding of the etiology of AD hinders the development of knowledge-based targeted therapeutics, various studies have demonstrated that alterations in the levels of specific neurotrophic factors (NTFs), such as brain-derived neurotrophic factor (BDNF) and ciliary neurotrophic factor (CNTF), are associated with the pathogenesis of neurodegenerative diseases such as AD and Parkinson’s disease (PD) [5,7,8,9,10,11,12,13]. These observations suggest that the sustained expression of neurotrophic factors may be useful for protecting neurons in the adult brain, and the regulatory system of a key molecule that can produce neurotrophic factors is a potential therapeutic target against neurodegenerative diseases.

Ras homolog protein enriched in brain (Rheb) is a member of the Ras superfamily containing Rheb1 and Rheb2 [14]. Rheb is expressed at high basal levels in the hippocampus, cerebral cortex, occipital pole, frontal lobe, and temporal lobe [15]. Rheb is biochemically activated by growth factors such as epithelial growth factor (EGF), fibroblast growth factor (FGF), glial cell line-derived neurotrophic factor (GDNF), CNTF, and BDNF and it is associated with neuronal growth, differentiation, aging, axonal regeneration, energy homeostasis, autophagy, and amino acid uptake [16,17]. It is well known that Rheb functions as a key activator of mammalian target of rapamycin complex 1 (mTORC1), and the deletion of Rheb could lead to reduced cortical thickness and defective myelination in the brain [18]. Rheb could negatively regulate autophagy [19], resulting in protein aggregation, which can be associated with the progression and severity of neurodegenerative diseases such as AD, PD, and Huntington disease (HD) [20,21,22,23]. However, there has been accumulating evidence of the neuroprotective effects of enhanced Rheb expression against neurodegenerative diseases including PD [11,24,25], AD [26,27], and spinal cord injury [28]. Interestingly, the hyperactivation of Rheb could protect hippocampal neurons via neurotrophic interactions between neurons and astrocytes against neurotoxic conditions in the hippocampus of the adult brain [5,26,29,30].

A cure for neurodegenerative diseases has not been developed; however, accumulating evidence suggests that Rheb may be considered as one of the possible treatment targets for neurodegenerative diseases due to its pleiotropic role in the production of various NTFs. Therefore, this study provides insight into the role of Rheb signaling pathways in neurodegenerative diseases and the beneficial effects of Rheb as a potential therapeutic agent against hippocampal neurodegeneration in the adult brain.

## 2. Characteristics of Rheb

Rheb proteins, monomeric proteins of approximately 21 kDa, are highly conserved during evolution, and their expression is found in yeast and humans [14]. Rheb consists of 184 amino acids; the N-terminal 169 amino acids make up the GTPase domain, and the 15 remaining C-terminal residues make up a highly variable region ending in a CAAX motif. The crystal structures of Rheb bound with GTP, GppNHp, or GDP have been determined [14]. The structure of Rheb is highly similar to that of other small GTPases, with a closer resemblance to those of Ras and Rap than to those of Rab5A and RhoA [14]. In addition, due to the unique structure of Rheb, Gln64 (corresponding to Gln61 in Ras) is buried in a hydrophobic core and cannot interact with either GTP or the catalytic active site. Another difference compared with Ras is that a conserved tyrosine residue (Tyr35) in Rheb (corresponding to Tyr32 in Ras) shields the phosphate moiety of GTP. These two unique structural features of Rheb suggest that the mechanism of GTPase in Rheb differs from that in Ras. Recent findings have clarified the role of Rheb in activating mTORC1 signaling at the lysosomal membrane [31,32]. Two different complexes interact with each other on the lysosomal membrane. One of the complexes contains mTORC1, which is localized in lysosomes by the action of a heterodimeric GTPase (Rag) [33,34]. Rag consists of RagA/RagC or RagB/RagD. The binding of Rag to the lysosomal membrane requires Ragulator, which consists of multiple proteins including p18, p14, and MP1. The other complex is tuberous sclerosis complex (TSC)/Rheb, which is localized in lysosomes via a farnesyl group on Rheb. The co-localization of Rheb and mTORC1 facilitates the activation of mTORC1 by Rheb. Further insights into the regulation of these lysosomal events can be gained from recent studies that investigated the signals affecting the localization of these proteins [35,36]. Insulin causes the dissociation of TSC and the release of TSC from lysosomes, resulting in the activation of Rheb [35]. On the other hand, amino acid starvation does not affect the lysosomal localization of TSC2. Since amino acid starvation affects mTORC1 localization, it is suggested that insulin signals through TSC/Rheb, while amino acids signal through Rag and mTORC1. However, Rag GTPase binds to TSC2, and this binding is stimulated by amino acid starvation [36]. Thus, further studies should reveal the interplay between two different signals that converge on the lysosomal membrane.

## 3. Rheb–mTOR Signaling in the Brain

Tuberous sclerosis (TS) disease, also called TSC, is a genetic disorder characterized by the growth of numerous noncancerous tumors in many parts of the body [37]. TSC1 and TSC2, intracellular molecules named after TSC, are associated with the activation of Rheb/mTOR signaling pathway [24,25]. The activity of Rheb is regulated by TSC2, which acts as a guanosine triphosphatase-activating protein (GAP) that enhances the hydrolysis of guanosine triphosphate (GTP) to guanosine diphosphate (GDP) by Rheb [24,38,39,40,41,42]. The heterodimer of the two proteins is a critical negative regulator of Rheb, resulting in the inhibition of mTOR [43], which is controlled by insulin. Insulin triggers the activation of the phosphoinositide 3-kinase (PI3K)/protein kinase B (Akt) pathway. Activated Akt then increases TSC2 phosphorylation at serine 939 and 981, leading to the dissociation of TSC1/2 [44].

It is well known that mTOR, a serine/threonine kinase that belongs to the family of PI3K-related kinases, acts as a central protein that controls cell growth and proliferation through transcriptional and translational mechanisms in response to various extracellular stimuli such as amino acids and growth factors [45]. Recently, a study demonstrated the requirement of intact F-actin dynamics for proper mTORC1 activation in response to netrin-1 in the axonal growth cones of tectal neurons [46]. mTORC1 is rapamycin-sensitive and contains regulatory-associated protein of mTOR (Raptor), mammalian lethal with SEC13 protein 8 (mLST8), and the 40 kDa proline-rich Akt substrate (PRAS40) [47,48]. The downstream targets of mTOR include two independent targets, i.e., ribosomal protein S6 kinase 1 (S6K1) and eukaryotic initiation factor 4E-binding protein 1 (4E-BP1), both of which play important roles in mRNA translation [49]. mTOR stimulates translational initiation through direct phosphorylation and activation of S6K1 [50] and 4E-BP1 [51]. mTOR has also emerged as an important regulator of various neurological processes including neuronal differentiation, morphogenesis, synaptic plasticity, learning, and memory [52,53]. The mTOR pathway has been found to adversely affect neural circuit formation. Furthermore, the mTOR pathway regulates axon length [54] and axon guidance in response to environmental signals [55]. Among neurons, local protein synthesis in synapses distant from the soma is mediated by mTOR and is critical for the formation of the neural circuit. The expression levels of synaptic proteins, such as Arc and synapsin, are increased by mTOR activation [56]. The hyperactivation of the mTOR pathway via the loss of *Pten* has been shown to increase glutamatergic and GABAergic signals [57]. In addition to neurons, the mTOR pathway has been found to regulate glial cells during neural circuit formation. Deletion of the core component of mTORC1 or mTORC2 (*Raptor* or *Rictor*) was reported to result in defective myelination and oligodendrocyte maturation [58]. mTORC1 activity is important for regulating the organism size and survival during embryonic development. The deficiency of Raptor leads to a reduced brain size in developing embryos due to impaired cell cycle progression and increased apoptosis [59]. Rheb knockout has been observed to cause embryonic lethality in mice. Moreover, mTOR has been reported to be critical for long-lasting forms of neuronal plasticity, such as long-term potentiation (LTP), long-term depression (LTD), and learning and memory, which require protein synthesis [53,60,61,62]. For example, learning transiently increases p-mTOR in the hippocampus [62,63]. The blockade of mTOR signaling with rapamycin impairs hippocampus-dependent learning in tasks such as inhibitory avoidance [64], and both voluntary [65,66] and forced exercise enhance learning in the same task [67]. A recent study showed that Rheb overexpression-mediated synaptic growth was morphologically and functionally different from that in the presence of the TSC mutant, indicating that TSC may be able to regulate the neuromuscular junction synapse independent of the Rheb–mTORC1 pathway [68]. In addition, Rheb augments the expression of NTFs such as BDNF, CNTF, and GDNF through the activation of mTORC1 in the brain [11,69]. NTFs support the cell growth, survival, synaptic plasticity, and differentiation of both developing and mature neurons [70]. On the other hand, Rheb activation inhibits the non-selective degradation of protein aggregation mediated by autophagy, resulting in cell death due to the accumulation of misfolded proteins [19]. Autophagy dysfunction can be implicated in the pathogenesis of neurodegenerative disorders including PD and AD [20,21,22,23]. However, it is still unclear whether the alteration of autophagic regulation by neuronal Rheb upregulation is involved in neurotoxicity or neuroprotection in the adult brain with neurodegenerative diseases. Therefore, further studies are needed to determine the connection between neuroprotection and Rheb-mediated autophagy in neurodegenerative diseases such as PD and AD.

## 4. Role of Rheb in Neurodegeneration in the Hippocampus

As mentioned previously, Rheb augments the expression of NTFs such as BDNF, CNTF, and GDNF through the activation of mTORC1 in the brain. NTFs regulate the development, maintenance, function, and plasticity of mature neurons. BDNF is essential for a basal level of adult hippocampal neurogenesis and the survival and integration of new-born neurons into the hippocampal circuitry [9,71,72]. BDNF also plays a crucial role in the early and late phases of LTP, the cellular substrate for learning and memory [9,71,72,73,74,75,76]. Altered BDNF functionality has been observed in different neurodegenerative diseases [77,78]. Several studies showed that the mRNA and protein expression levels of BDNF and its full-length receptor tropomyosin receptor kinase B (TrkB) are decreased in the hippocampus and neocortex of AD postmortem brains and in the substantia nigra of PD patients [7,79,80,81]. BDNF administration has been shown to improve learning and memory in demented animals [82]. In addition, BDNF has been observed to have neuroprotective effects against β-amyloid (Aβ) and tau toxicity in AD models [83,84].

GDNF binds to its co-receptor alpha1 (GFRα1) and can promote the survival of different neuronal populations in the central and peripheral nervous systems [85]. The Ras/MAPK and PI3K/Akt cascades can be activated when GDNF binds to its main receptor, which consists of the ligand-binding component GFR*α* and the tyrosine kinase RET [86,87]. Several studies on the ability of GDNF to maintain mitochondrial activity have been conducted. By using a mouse model of PD, Meka et al. showed that GDNF could improve impaired mitochondrial function by activating the NF-*κ*B transcription factor, mediated by the RET kinase through the PI3K pathway [88]. The GDNF–GFRα1 complex is essential for proper hippocampal circuit development. GDNF–GFRα1 signaling contributes to synapse development and the maturation of hippocampal neurons [89]. The GDNF–GFRα1 complex was observed to promote dendritic growth and postsynaptic differentiation in cultured hippocampal neurons through neural cell adhesion (NCAM) signaling [89]. The overexpression of GDNF in hippocampal astrocytes induced the recovery of spatial cognitive abilities in aged impaired rats. Several studies have shown that GDNF can enhance motor functions in aged rats and non-human primates, which may be associated with dopaminergic induction and regeneration of the nigrostriatal pathway [90,91,92,93,94]. Furthermore, GDNF is known to promote neuronal health and has neural regenerative properties [95]. Low GDNF levels were reported in patients with AD and in 3xTg AD mice [96,97]. In addition, GFRα1 deficiency has been observed in the brain of AD patients [98]. GDNF overexpression in astrocytes was found to exhibit neuroprotective effects through the upregulation of BDNF production, resulting in the preservation of learning and memory in 3xTg AD mice [99]. In addition, the upregulation of GDNF in activated astrocytes after brain injury is thought to play an active role in neuronal survival and plasticity [100]. Activated astrocytes have been observed to upregulate NTFs, antioxidants, and other key molecules, all of which support neuronal and oligodendrocyte survival as well as tissue repair [101]. These results suggest that the crosstalk between neurons and astrocytes is a potential neuroprotective mechanism of NTFs in neurodegenerative diseases.

CNTF belongs to the interleukin-6 family of cytokines and is expressed mainly in astrocytes, whereas CNTF receptor α subunit (CNTFRα) is expressed predominantly in neural progenitor cells and hippocampal neurons [102]. CNTF has potent effects on the development and maintenance of the nervous system, inducing neuronal survival and differentiation by stimulating gene expression in sensory, sympathetic, and motor neurons [103]. In addition, the neuroprotective properties of CNTF demonstrate that it is a survival factor for sympathetic, sensory, and hippocampal neurons. CNTF could promote neural stem cell division directly through its receptor [104]. It has been reported that CNTF protects neurons from degeneration arising from multiple etiologies [105,106]. In a previous study, CNTF contributed to the full recovery of cognitive functions associated with the stabilization of synaptic protein levels in the Tg2576 AD mouse model [107]. These results indicate the potential of CNTF as a therapeutic agent for treating neurodegenerative diseases. Taken together, increasing evidence suggests that a lack of trophic support may contribute significantly to neurodegeneration, and NTFs have emerged as promising therapeutic agents for neurodegenerative diseases [108]. Nevertheless, the clinical utility of systemic BDNF, GDNF, and CNTF is limited by poor blood–brain barrier (BBB) permeability, a short half-life, and off-target effects [109,110].

Rheb has been reported to increase the levels of acetylcholine and total choline in the adult rat brain, which are important for maintaining cognitive functions in AD brains [5]. Axon degeneration and synapse and dendritic spine loss have been observed in brains as a result of aging and neurodegenerative diseases [111,112,113]. A decrease in synaptic connectivity is considered to be a major cause of altered mood and impaired perception and cognition in neurodegenerative diseases. Several studies have demonstrated the role of Rheb–mTOR signaling in axon elongation and synaptic morphogenesis [114,115]. Akt–Rheb–mTORC1 signaling can enhance not only the axon length but also the axon number per neuron and the number of neurons having multiple axons [116]. In addition, Rheb activation can enhance dendritic morphogenesis and synaptic integration in the subventricular zone [117]. TSC2–Rheb–mTOR signaling regulates the ephrin–Eph receptor system to mediate axon guidance in the visual system [118]. Furthermore, long-term synaptic plasticity, learning, and memory rely on de novo protein synthesis. Rheb–mTOR signaling is considered an important mechanism for the maintenance of LTP and learning and memory functions [60,61]. Rheb has been reported to interact with beta-secretase 1 (BACE1) in the mouse brain, resulting in the inhibition of BACE1 activity and the reduction of Aβ generation [119]. Furthermore, the deletion of Rheb could lead to deficits in spatial memory functions, a behavioral hallmark of AD progression [119]. In the brain of patients with AD, the levels of Rheb are significantly downregulated compared with its levels in a normal healthy brain [23].

On the other hand, Rheb can be an issue in neurodegenerative disease progression. Several studies indicated that TSC–Rheb–mTOR signaling could regulate aggresome formation, contributing to the disposal of misfolded proteins via the proteasome and autophagy system. Autophagy failure can trigger neuronal cell death in several ways, depending on where the defect is along the pathway. When proteolytic clearance steps are compromised, autolysosomes/lysosomes accumulate mutant and oxidized proteins, protein oligomers and aggregates, damaged organelles, and other incompletely digested products, some of which increase the permeability of lysosomal membranes and cause hydrolases to be released into the cytoplasm, in some cases even from otherwise intact lysosomes [112]. Autophagy-related pathology has been noted in late-onset neurodegenerative diseases including AD, PD, amyotrophic lateral sclerosis (ALS), and HD [23]. mTORC1 negatively controls the initiation of autophagy, contributing to the formation of pathological protein aggregates. Previous studies [120,121] showed that mTOR activation could downregulate Aβ clearance by inhibiting autophagy functions with the suppression of several signaling pathways including PI3K/Akt, glycogen synthase kinase 3 (GSK-3), AMP-activated protein kinase (AMPK), and insulin/insulin-like growth factor 1 (IGF-1) [122]. In addition, the activation of mTOR contributes to aberrant hyperphosphorylated tau [123]. The alteration of mTOR activity may be correlated with neurological symptoms such as epilepsy, mental retardation, autism, and brain tumors [61,124,125,126]. These findings suggest that Rheb–mTORC1 signaling may be associated with the onset and progression of neurodegenerative diseases.

Although the Rheb–mTOR signaling pathway may act as a double-edged sword in neurodegeneration, it is a central regulator of NTFs such as BDNF, CNTF, and GDNF and thus is a potential therapeutic target for neurodegenerative diseases. Therefore, studies that elucidate the role of the Rheb–mTOR signaling pathway in neurodegenerative diseases are of great importance.

## 5. Mutation of Rheb Leading to Constitutive Activation

The expression levels of growth-associated proteins are lower in adulthood than in the process of development. Consequently, the regenerative capacity of mature neurons is limited in the aging brain [127]. As described previously, Rheb–mTOR signaling is a key regulator of various mechanisms associated with neuronal survival and regeneration. Accordingly, several studies have identified Rheb mutations that lead to constitutive activation (Table 1). The mutation of serine to histidine at position 16 of Rheb [Rheb(S16H)] has been found to exhibit gain-of-function properties, resulting in highly activated mTOR signaling in TSC1/2-overexpressing cells [128]. Our previous studies showed that the overexpression of Rheb(S16H) could significantly increase the levels of NTFs such as GDNF, CNTF, and BDNF in the adult brain [5,11,26,29,69]. The mutation of tyrosine to asparagine at position 35 of Rheb [Rheb(Y35N)] in renal cell carcinoma has been reported to cause mTORC1 hyperactivation by increasing the resistance to TSC2 GAP activity, resulting in the hyperproliferation of tumor cells [129]. Another study identified the mutation of glycine to alanine at position 63 of Rheb [Rheb(G35A)], which could stimulate the phosphorylation of S6 kinase more strongly compared wild-type Rheb in HeLa cells [130]. The mutation of glutamine to leucine at position 64 of Rheb [Rheb(Q64L)] was found to strongly induce oncogenic transformation in chicken embryonic fibroblast cultures. Cells with Rheb(Q64L) overexpression could promote the constitutive phosphorylation of the ribosomal proteins S6K and 4E-BP1 [131] and exhibit increased GTP loading and partial resistance to TSC–GAP [132]. Another study identified specific Rheb hyperactive mutations in four different residues: (a) V17G and V17A, (b) S21G and S21I, (c) K120R, (d) N153S and N153T [133]. In comparison with wild-type Rheb, these hyperactive mutations of Rheb conferred resistance to canavanine toxicity. The GTP-binding affinity of these mutations was closely similar to that of wild-type Rheb, whereas the GDP-binding affinity was greatly reduced. In our previous study, we demonstrated the neuroprotective potential of the constitutively active forms of Rheb with mutations such as Rheb(S16H) and Rheb(N153T) in a neurotoxin-induced animal model of PD [24]. In particular, Rheb(S16H) upregulation by adeno-associated virus serotype 1 (AAV1) administration in nigral dopaminergic neurons significantly increased the activation of mTORC1, resulting in greater neuroprotective effects compared with Rheb(N153T) upregulation in the animal model of PD [24]. In addition, our studies demonstrated that the upregulation of neuronal Rheb(S16H) could protect neurons against neurodegeneration and promote axonal regrowth in the nigrostriatal dopamine system and hippocampus of the adult brain [11,24,25,30,69,134]. The activation of mTORC1 induced by Rheb(S16H) transduction of hippocampal neurons was found to result in BDNF production, which protected against thrombin-induced neurotoxicity in the rat hippocampus [5]. In the same manner, AAV1–Rheb(S16H) transduction in the hippocampus of transgenic AD mice prevented cognitive function impairment [26,29].

## 6. Mechanisms of Rheb(S16H)-Induced Neuroprotection in the Hippocampus of the Adult Brain

To date, the upregulation of neuronal Rheb(S16H) has demonstrated some beneficial effects in the hippocampus of the adult brain under neurotoxic conditions. Our previous studies demonstrated that neuronal transduction with AAV1–Rheb(S16H) prevented thrombin-induced neuronal death in the hippocampus of the adult rat brain [5,29]. Similarly, previous studies reported that neuronal transduction of Rheb(S16H) could protect dopaminergic neurons in the substantia nigra of mice against neurotoxin-induced dopamine neuronal death [11,24,25,69] and prothrombin kringle-2 (pKr-2)-induced neurotoxic inflammation [30]. AAV1–Rheb(S16H) transduction was found to have preventive effects on LTP impairment and cognitive decline in 5XFAD mice, which represent a transgenic mouse model of AD carrying five mutations associated with early-onset familial Alzheimer’s disease (FAD) [26]. These neuroprotective effects could be mediated by the stimulation of mTORC1-related neuronal BDNF production following AAV1–Rheb(S16H) administration, which might occur regardless of the levels of neuroinflammatory molecules. In addition, we recently found that neuronal transduction with AAV1–Rheb(S16H) could increase the expression of GDNF in both hippocampal neurons and astrocytes via autocrine and paracrine BDNF–TrkB signaling [29,135]. Similarly, previous studies reported that the expression of neuronal Rheb(S16H) could protect dopaminergic neurons through the elevation of GDNF and BDNF expression in the substantia nigra against neurotoxin-induced dopamine neuronal death [69] and pKr-2-induced neurotoxic inflammation [30]. Furthermore, Rheb(S16H) upregulation could induce the upregulation of cAMP response element-binding protein (CREB) phosphorylation in dopamine neurons, which may be associated with the synthesis of GDNF and BDNF. In the hippocampus of the adult brain, AAV1–Rheb(S16H) transduction could induce sustained increases in the levels of full-length TrkB and CNTFRα in reactive astrocytes and hippocampal neurons, respectively [29]. In addition, we demonstrated that neuronal BDNF produced by the transduction of hippocampal neurons with Rheb(S16H) could induce reactive astrocytes, which could lead to CNTF production through the activation of astrocytic TrkB and the upregulation of neuronal BDNF and astrocytic CNTF, resulting in synergistic effects on the survival of hippocampal neurons in vivo [29]. In 5XFAD mice, AAV1–Rheb(S16H) transduction was found to have preventive effects on LTP impairment and cognitive decline [26]. These beneficial effects may be attributed to the interaction between neurons and astrocytes promoted by multiple NTFs induced by Rheb(S16H) transduction of hippocampal neurons, which result in neuroprotection in the hippocampus of 5XFAD mice.

**Figure 1:**
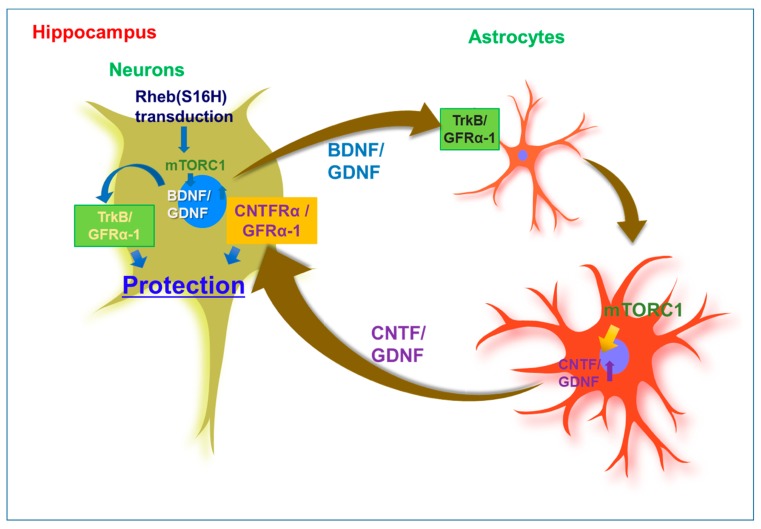
Mechanisms of neuroprotection through neuronal upregulation of mutated ras homolog protein enriched in brain [Rheb(S16H)] in the hippocampus of the adult brain. Upregulation of Brain-derived neurotrophic factor (BDNF) and glial cell line-derived neurotrophic factor (GDNF) by neuronal transduction with adeno-associated virus serotype 1 (AAV1)–Rheb(S16H) can additionally contribute to the activation of BDNF/TrkB and GDNF/GFRα-1 signaling pathways, respectively, in astrocytes via the interactions between neurons and astrocytes in the adult hippocampus, resulting in the production of astrocytic ciliary neurotrophic factor (CNTF) and GDNF for hippocampal neuroprotection. These observations suggest that Rheb(S16H) transduction of hippocampal neurons may be a good strategy to protect adult neurons in the hippocampus in vivo.

Astrocytes are the most abundant cells in the brain and play an important role in the homeostatic maintenance of physiological environments, such as ion concentration and pH in the central nervous system [136,137]. Although previous studies indicated that astrocyte activation is involved in neurodegeneration through inflammatory responses in the adult brain [138,139], reactive astrocytes may have beneficial effects such as increased neuronal survival, growth, and activity through barrier function to restrict tissue damage and neuroinflammation [137,140,141]. In addition, they have been found to produce NTFs such as GDNF and CNTF in animal models of neurodegenerative diseases [142,143,144]. A study reported that the loss of astrocytes in the brain of GFAP-knockout mice resulted in a larger infarct area induced by ischemic brain injury following middle cerebral artery occlusion [145]. Overall, these findings suggest that astrocytes are important for maintaining a normal neural system in the adult brain. Furthermore, the induction of reactive astrocytes, which can protect against neurodegeneration in patients with neurodegenerative diseases such as AD and PD, was observed to support neuronal survival and functional maintenance in vivo. In previous studies, morphological changes in astrocytes and microglia were clearly observed in the Rheb (S16H)-treated hippocampus of the adult brain [26,29,135]. Moreover, the levels of Aβ were significantly reduced after AAV1–Rheb(S16H) transduction in the hippocampus of 5XFAD mice. These observations suggest that AAV1–Rheb(S16H) transduction of hippocampal neurons could strengthen the neuroprotective system through the stimulation of mTORC1-mediated neurotrophic interactions involving BDNF, CNTF, and GDNF between neurons and astrocytes in the hippocampus of the adult brain, resulting in neuroprotection in the hippocampus in vivo under various neurodegenerative conditions (Figure 1).

## 7. Conclusion

The current therapies for AD and PD are initially effective, alleviating the main symptoms of these diseases. However, disease progression is not prevented. In contrast to the current treatments, Rheb(S16H) transduction of adult neurons may provide a novel therapeutic option. Our recent studies suggest that Rheb(S16H) transduction of hippocampal neurons could stimulate NTFs production, strengthening the neuroprotective system in the hippocampus of the adult brain and reducing neurodegeneration. Although further studies are needed to determine the clinical relevance of the AAV1–Rheb(S16H) transduction approach for neurodegenerative diseases, this approach may be a useful strategy for protecting hippocampal neurons in the lesioned brain, and its effects may be beneficial for patients with neurodegenerative diseases such as AD.

## Figures and Tables

**Table 1:** List of mutations associated with the constitutive activation of Rheb.

Amino Acid Position	Mutation	Cell Type	Reference
**16**	S16H	TSC1/2-overexpressing HEK293T	[128]
**17**	V17G, V17A	fission yeast	[133]
**21**	S21G and S21I	fission yeast	[133]
**35**	Y35N	renal cell carcinoma	[129]
**63**	G63A	HeLa cells	[130]
**64**	Q64L	chicken embryonic fibroblasts	[131]
**120**	K120R	fission yeast	[133]
**153**	N153T	fission yeast	[128,133]
		TSC1/2-overexpressing HEK293T	[128]
